# Microsatellite instability in sporadic colorectal cancer is not an independent prognostic factor

**DOI:** 10.1038/sj.bjc.6690676

**Published:** 1999-09

**Authors:** S Salahshor, U Kressner, H Fischer, G Lindmark, B Glimelius, L Påhlman, A Lindblom

**Affiliations:** Department of Molecular Medicine, Karolinska Institute, Stockholm, S171 76, Sweden; Department of Surgery, Ueddevalla Hospital, Sweden; Department of Surgery, University Hospital, University of Umeå, Sweden; Department of Oncology, Radiology and Clinical Immunology, Surgery, University Hospital, University of Uppsala, Sweden

**Keywords:** microsatellite instability (MSI), prognosis, colorectal cancer

## Abstract

Hereditary non-polyposis colorectal cancer (HNPCC) is linked to an inherited defect in the DNA mismatch repair system. DNA from HNPCC tumours shows microsatellite instability (MSI). It has been reported that HNPCC patients have a better prognosis than patients with sporadic colorectal cancer. We examined whether the presence of MSI in a series of unselected colorectal tumours carries prognostic information. In a series of 181 unselected colorectal tumours, 22 tumours (12%) showed MSI. Survival analysis at 5–10 years follow-up showed no statistically significant difference in prognosis between MSI-positive and -negative tumours. Our results suggest that the MSI phenotype as such is not an independent prognostic factor. © 1999 Cancer Research Campaign


*British Journal of Cancer* (1999) **81**(2), 190–193
© 1999 Cancer Research Campaign
Article no. bjoc.1999.0676

### Microsatellite instability in sporadic colorectal cancer is not an independent prognostic factor

**S Salahshor[1], U Kressner[2], H Fischer[1], G Lindmark[3], B Glimelius[4], L Påhlman[5] and A Lindblom[1]**

[1]Department of Molecular Medicine, Karolinska Institute, S171 76 Stockholm, Sweden; [2]Department of Surgery, Ueddevalla Hospital, Sweden; [3]Department of Surgery, University Hospital, University of Umeå, Sweden; [4]Department of Oncology, Radiology and Clinical Immunology, and [5]Surgery, University Hospital, University of Uppsala, Sweden

**Summary** Hereditary non-polyposis colorectal cancer (HNPCC) is linked to an inherited defect in the DNA mismatch repair system. DNA from HNPCC tumours shows microsatellite instability (MSI). It has been reported that HNPCC patients have a better prognosis than patients with sporadic colorectal cancer. We examined whether the presence of MSI in a series of unselected colorectal tumours carries prognostic information. In a series of 181 unselected colorectal tumours, 22 tumours (12%) showed MSI. Survival analysis at 5–10 years follow-up showed no statistically significant difference in prognosis between MSI-positive and -negative tumours. Our results suggest that the MSI phenotype as such is not an independent prognostic factor.

© 1999 Cancer Research Campaign

**Keywords:** microsatellite instability (MSI); prognosis; colorectal cancer

Colorectal cancer (CRC), as all other cancers, seems to be genetically unstable. This instability can be of two kinds: chromosomal instability (CIN) or microsatellite instability (MSI) (Lengauer et al, 1997). Microsatellites are simple repeats, often a dinucleotide, on non-coding regions of DNA, which could be located within genes or in between genes (Weber and May, 1989). MSI was first described in a set of unselected CRC (Ionov et al, 1993; Thibodeau et al, 1993) and in hereditary non-polyposis colorectal cancer (HNPCC) (Aaltonen et al, 1993; Lindblom et al, 1993). HNPCC is caused by germ-line mutations in genes involved in DNA mismatch repair (MMR) (Kinzler and Vogelstein, 1996). MSI can be detected in more than 90% of HNPCC tumours (Aaltonen et al, 1993; Tannergård et al, 1997). This increased mutation rate is obtained from a defective MMR. HNPCC patients have a better prognosis than sporadic CRC cases (Fujita et al, 1996; Sankila et al, 1996; Myrhoj et al, 1997; Percesepe et al, 1997). Approximately 12–17% of the unselected tumours also show MSI (Aaltonen et al, 1993; Ionov et al, 1993; Thibodeau et al, 1993). Very few mutations in MMR genes have been found in unselected MSI-positive tumours. However, using immunohistochemistry, it has been suggested that the MSI phenotype even in unselected MSI-positive tumours involves lack of MMR gene function. In 14 out of 15 MSI-positive tumours tested, the expression of either hMLH1 or hMSH2 was lacking (Thibodeau et al, 1996; Dietmaier et al, 1997). A possible mechanism for this can be inactivation of the hMLH1 gene by hypermethylation of its promotor (Kane et al, 1997; Herman et al, 1998). In accordance with this, patients with sporadic or unselected CRC displaying MSI have been suggested to have a better prognosis than those without (Lothe et al, 1993; Thibodeau et al, 1993; Bubb et al, 1996). In the current study we wanted to explore further the relations between MSI status and prognosis in unselected CRC, in a large consecutive series of CRC tumours obtained from two surgery clinics, and previously studied for prognostic correlation to various parameters.

*Received 13 October 1998*
*Revised 22 March 1999*
*Accepted 30 March 1999*

*Correspondence to:* A Lindblom

### MATERIALS AND METHODS

### Patients

One hundred and eighty-one unrelated patients with CRC treated at the Departments of Surgery in Uppsala and Falun between 1988 and 1992 were included in the study. Adjuvant preoperative radiotherapy was given to 28 of 62 patients with rectal cancer, and one patient with colon cancer had post-operative adjuvant chemotherapy. The tumours were graded according to the WHO classification system (Morson and Sobin, 1976), and staged according to the Dukes' classification system (Dukes and Bussey, 1958). Clinicopathological characteristics are given in Table 1.

### DNA extraction

The samples were frozen and stored at –70°C prior to DNA extraction. DNA was prepared by proteinase-K digestion and phenol–chloroform extraction according to standard procedures.

### Microsatellite analysis

Dinucleotide repeats D22S428, D22S272, PDGF and mononucleotide repeats transforming growth factor beta receptor 2 (TGF-β-R II), BAT-26, BAT-25 were used to type all tumours with normal DNA available. For the 86 tumours where no normal DNA was available, only the three mononucleotide markers were used. Primers specific for each locus were used to amplify the repeat and short flanking sequences in template DNA by polymerase chain reaction (PCR). One of the primers was labelled with $\gamma[^{32}P]$dCTP prior to amplification. PCR was performed on normal

**Table 1** MSI status in colorectal cancer and its relation to age, gender, tumour stage, tumour differentiation and tumour localization

| | Number of case | Number of MSI+ (%) tumours | *P*-value | Number of cancer related deaths | |
|---|---|---|---|---|---|
| | | | | MSI+ (%) | MSI– (%) |
| Age | | | NS | | |
| ≤ 70 | 83 | 6 (7) | | 1 (17) | 29 (38) |
| > 70 | 98 | 16 (16) | | 4 (25) | 34 (41) |
| Gender | | | NS | | |
| Male | 78 | 14 (10) | | 2 (14) | 31 (48) |
| Female | 103 | 8 (14) | | 3 (3) | 32 (34) |
| Tumour stage | | | NS | | |
| A | 28 | 4 (14) | | 0 (0) | 3 (12) |
| B | 92 | 15 (16) | | 3 (20) | 17 (22) |
| C | 37 | 3 (8) | | 2 (66) | 19 (56) |
| D | 24 | 0 | | 0 (0) | 24 (10) |
| Tumour differentiation[a] | | | NS | | |
| Good | 25 | 2 (8) | | 0 (0) | 4 (17) |
| Moderate | 120 | 16 (13) | | 4 (25) | 40 (38) |
| Poor | 36 | 4 (11) | | 1 (25) | 19 (59) |
| Tumour localization | | | 0.075 | | |
| Proximal colon | 119 | 19 (15) | | 4 (21) | 27 (27) |
| Distal colon (Including rectum) | 62 | 3 (5) | | 1 (33) | 36 (61) |

[a]According to the WHO classification. NS, not significant.

**Table 2** Univariate analyses showing the MSI status, gender, age, Dukes' stages and tumour differentiation on prognosis of 181 patients

| Variable | *P*-value | RH | CI |
|---|---|---|---|
| MSI | | | |
| MSI+ | | 1.0 | Ref |
| MSI– | 0.20 | 1.81 | 0.73–4.44 |
| Gender | | | |
| Male | | 1.0 | Ref |
| Female | 0.10 | 0.68 | 0.42–1.08 |
| Age | 0.10 | 1.02 | 0.99–1.08 |
| Dukes' | | | |
| A | | 1.0 | Ref |
| B | 0.15 | 2.43 | 0.72–8.14 |
| C | 0.001 | 7.61 | 2.26–25.5 |
| D | 0.0001 | 47.7 | 13.9–163.2 |
| Tumour differentiation | | | |
| Good | | 1.0 | Ref |
| Moderate | 0.048 | 2.8 | 1.0–2.86 |
| Poor | 0.002 | 5.4 | 1.82–15.8 |

RH, relative hazard.

and tumour DNA using 50 ng of purified genomic DNA in a final volume of 20 µl. PCR conditions were 95°C for 2 min followed by 35 cycles (94°C for 45 s, 50–60°C for 45 s and 70°C for 1 min), and a final elongation at 70°C for 7 min. Reactions were resolved on urea-formamide polyacrylamide gel and exposed to film.

### MSI analysis

Criteria used for MSI in our material are as follows: for samples, where both normal and tumour material were available, six markers were used and MSI defined as an alteration in at least three out of six markers. In 86 samples where constitutional DNA was not available, MSI was defined as an alteration in at least two loci of three tested markers. Tumours showing one alteration but not fulfilling the criteria above called MSI low (MSI-L) (Boland et al, 1998; Perucho, 1999) were considered as MSI-negative tumours in the analysis.

### Statistical analysis

Cause-specific survival analysis (death from colorectal cancer) was analysed with the Cox proportional hazard model. Survival curves were constructed using the Kaplan–Meier method, and differences tested using the log-rank test. The $\chi^2$ test was used to test for differences in distribution among groups. Correlation coefficients were calculated when testing correlation among groups (Cox, 1972; Peto et al, 1977). The statistical software Statistica (Statsoft Inc® version 5.0) was used for the analyses.

### RESULTS

In 93 tumours with normal DNA available, we found 11 tumours (12%), with MSI according to the criteria of at least three of six markers used, and in tumours without normal DNA available, 11 tumours (12%) showed MSI according to the criteria of at least two of three mononucleotide markers showing additional bands. Thus, in accordance with previous studies we detected 12% MSI positive tumours in this unselected material of CRC. The mean age of onset of CRC was 69 years (range 39–91).

As expected, of the 22 MSI-positive tumours, the vast majority, 18 (81%), was found in the proximal colon. There was no correlation between MSI status and age or gender (Table 1). None of the 24 Dukes' D tumours were shown to be MSI-positive. The MSI-positive tumours seem to be of a generally earlier stage. There were no statistically significant differences between MSI-positive and MSI-negative tumours compared by each stage (Table 1).

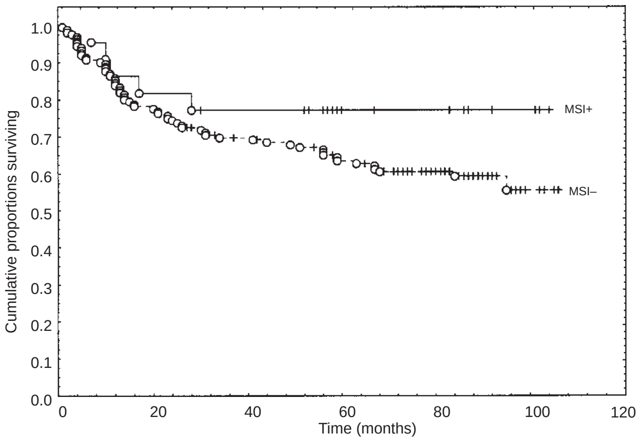

**Figure 1** Life-table plots for all patients (Dukes' stages A–D). MSI-positive (—) versus MSI-negative (- - -). O indicates code for complete responses, and + code for censored responses (i.e. patients who are alive or who have died from other causes than cancer)

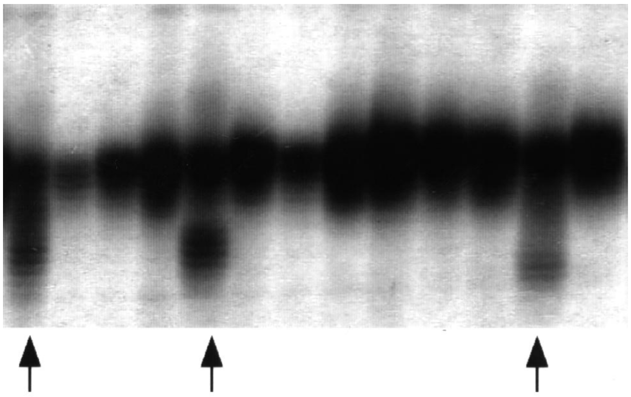

**Figure 2** Microsatellite analysis with BAT-26 marker. MSI-positive tumours have been indicated by arrows

At follow-up, 68 patients (39%) had died from cancer, or from other causes, but with a known tumour burden. The median survival time of the living patients was 87 months (range 51–106). Univariate survival analyses showed, as expected, a very strong correlation between Dukes' stage and prognosis, and a weaker but statistically significant correlation between tumour differentiation and prognosis (Table 2).

Survival analysis revealed no statistically significant difference in prognosis between MSI-positive and MSI-negative cases (Table 2), although a trend towards better survival for MSI-positive cases was observed (Figure 1). Survival analysis using Cox proportional hazard model confirmed the lack of significant correlation between MSI-positive tumours and prognosis (data not shown).

### DISCUSSION

Our result did not show a significant correlation between MSI-positive unselected colorectal tumours and good prognosis, compared to previous studies (Lothe et al, 1993; Thibodeau et al, 1993; Bubb et al, 1996). In this study, we used both mononucleotide markers and dinucleotide markers, including BAT-26, to test for MSI status. Previous studies mostly used different numbers of dinucleotide markers. Thibodeau et al (1993) used the criteria one or more alterations out of four markers, and Lothe et al (1993) used two or more alterations out of seven markers. Bubb et al used one or more alterations out of four dinucleotide markers, plus BAT-26. BAT-26, a quasimonomorphic marker (Figure 2), has been shown to be sufficient alone to give the MSI status of tumours even without normal DNA available (Bocker et al, 1997; Hoang et al, 1997; Zhou et al, 1998). However, in the study by Bubb et al (1996) BAT-26 was altered in only 58% of the tumours having at least one alteration found with the other four markers. Besides, BAT-26 alone identified three additional tumours in the latter study. Since this report indicated that there could be tumours showing MSI with dinucleotide markers but not BAT-26, we used additional dinucleotide markers (D18S70, D18S461, D18S58, D18S485, D18S483, D18S470, S18S1145, D18S57, D18S66) for the 93 tumours with normal DNA available. This test identified one more tumour to score as MSI-positive, because of dinucleotide markers, while BAT-26 was negative. It also showed that two tumours scored MSI-positive because of alterations in mononucleotide markers should have been MSI negative if only dinucleotide markers were used. It is possible that mono- and dinucleotide markers to some extent will identify different tumours as MSI-positive, but this does not explain the lack of statistically significance results obtained in our study.

We also found seven tumours expressing a low degree of MSI (MSI-L). Five of those had an alteration in one out of three mononucleotide markers and, the other two had one and two alterations, respectively, in six markers.

To test if our criteria for MSI were too stringent, we included the seven MSI-L tumours among the MSI-positive in a separate survival analysis. The correlation obtained was even less (data are not shown), indicating that the lack of correlation to prognosis was not dependent on too strict criteria used for typing a tumour as MSI.

Bubb et al carried out the survival analysis on 169 patients and the hazard ratio of patients with tumours showing MSI to those without was estimated to be 0.39 (Bubb et al, 1996). Lothe et al who studied 238 tumours using univariate cause specific (death by colorectal cancer) analysis found a significant association between MSI-positive and prolonged survival, the estimated hazard ratio was 0.3 (Lothe et al, 1993). Thibodeau et al also used univariate analysis of 86 patients with stage A to D colorectal cancer and found a correlation between MSI positive and overall survival ($P = 0.02$) (Thibodeau et al, 1993). Relative hazard estimated in our material was 0.55.

A correlation between Dukes' stage and MSI status in one set of tumours could give false significance. In Lothe's and Thibodeau's studies the significance was lost when Dukes' stage was corrected for in the analysis. However, in the Bubb study, where the significance was highest, there was no correlation between Dukes' stage and MSI status. In our study there was no significant difference between MSI-positive and MSI-negative tumours, if compared for each stage. Thus, the differences in prognosis seen in Figure 1, might be related to tumour stage at diagnosis in the MSI-positive tumours. It is possible that this tendency to a lower tumour stage at diagnosis might be related to a less malignant clinical courses. One explanation for this could be a more efficient immune defence in this group of patients.

In conclusion, although our results suggest that the presence of MSI indicates a weak favourable clinical courses, in a series of consecutive unselected CRC, MSI status is not an independent prognostic factor.

© 1999 Cancer Research Campaign

### ACKNOWLEDGEMENTS

This study was supported by the Swedish Cancer Society, the Nordic Cancer Union and Cancer Foundation in Stockholm.

### REFERENCES

Aaltonen L, Peltomaki P, Leach F, Sistonen P, Pylkkanen L, Mecklin J, Jarvinen H, Powell S, Jen J and Hamilton S (1993) Clues to the pathogenesis of familial colorectal cancer. *Science* **260**: 812–816

Bocker T, Diermann J, Friedl W, Gebert J, Holinski-Feder E, Karner-Hanusch J, Von Knebel-Doebertiz M, Koelble K, Moeslein G, Schackert H-K, Wirtz H-C, Fishel R and Ruschoff J (1997) Microsatellite instability analysis: a multicenter study for reliability and quality control. *Cancer Res* **57**: 4739–4743

Boland CR, Thibodeau SN, Hamilton SR, Sidransky D, Eshleman JR, Burt RW, Meltzer SJ, Rodriguez-Bigas MA, Fodde R, Ranzani GN and Srivastava S (1998) A National Cancer Institute Workshop on Microsatellite Instability for cancer detection and familial predisposition: development of international criteria for the determination of microsatellite instability in colorectal cancer. *Cancer Res* **58**: 5248–5257

Bubb VJ, Curtis LJ, Cunningham C, Dunlop MG, Carothers AD, Morris RG, White S, Bird CC and Wyllie AH (1996) Microsatellite instability and the role of hMSH2 in sporadic colorectal cancer. *Oncogene* **12**: 2641–2649

Cox D (1972) Regression models and life-tables (with discussion) *J Roy Stat Soc* **34**: 187–220

Dietmaier W, Wallinger S, Bocker T, Kullmann F, Fishel R and Ruschoff J (1997) Diagnostic microsatellite instability: definition and correlation with mismatch repair protein expression. *Cancer Res* **57**: 4749–4756

Dukes C and Bussey H (1958) The spread of rectal cancer and its effect on prognosis. *Br J Cancer* **12**: 309–320

Fujita S, Moriya Y, Sugihara K, Akasu T and Ushio K (1996) Prognosis of hereditary nonpolyposis colorectal cancer (HNPCC) and the role of Japanese criteria for HNPCC. *Jpn J Clin Oncol* **26**: 351–355

Herman JG, Umar A, Polyak K, Graff JR, Ahuja N, Issa J-PJ, Markowitz S, Willson JKV, Hamilton SR, Kinzler KW, Kane MF, Kolodner RD, Vogelstein B, Kunkel TA and Baylin SB (1998) Incidence and functional consequences of hMLH1 promoter hypermethylation in colorectal carcinoma. *Proc Natl Acad Sci USA* **95**: 6870–6875

Hoang J, Cottu P, Thuille B, Salmon R, Thomas G and Hamelin R (1997) BAT-26, an indicator of the replication error phenotype in colorectal cancers and cell lines. *Cancer Res* **57**: 300–303

Ionov Y, Peinado MA, Malkhosyan S, Shibata D and Perucho M (1993) Ubiquitous somatic mutations in simple repeated sequences reveal a new mechanism for colonic carcinogenesis. *Nature* **363**: 558–561

Kane MF, Loda M, Gaida GM, Lipman J, Mishra R, Goldman H, Jessup JM and Kolodner R (1997) Methylation of the hMLH1 promoter correlates with lack of expression of hMLH1 in sporadic colon tumors and mismatch repair-defective human tumor cell lines. *Cancer Res* **57**: 808–811

Kinzler KW and Vogelstein B (1996) Lessons from hereditary colorectal cancer. *Cell* **87**: 159–70

Lengauer C, Kinzler KW and Vogelstein B (1997) Genetic instability in colorectal cancers. *Nature* **386**: 623–627

Lindblom A, Tannergard P, Werelius B and Nordenskjold M (1993) Genetic mapping of a second locus predisposing to hereditary non-polyposis colon cancer. *Nat Genet* **5**: 279–2782

Lothe RA, Peltomaki P, Meling GI, Aaltonen LA, Nystrom-Lahti M, Pylkkanen L, Heimdal K, Andersen TI, Moller P, Rognum TO and et al (1993) Genomic instability in colorectal cancer: relationship to clinicopathological variables and family history. *Cancer Res* **53**: 5849–5852

Morson B and Sobin L (1976) Histological typing of intestinal tumors. In: *International Histological Classification of Tumours.* WHO, Geneva

Myrhoj T, Bisgaard ML, Bernstein I, Svendsen LB, Sondergaard JO and Bulow S (1997) Hereditary non-polyposis colorectal cancer: clinical features and survival. Results from the Danish HNPCC register. *Scand J Gastroenterol* **32**: 572–576

Percesepe A, Benatti P, Roncucci L, Sassatelli R, Fante R, Ganazzi D, Bellacosa A, Genuardi M, Neri G, Viel A and Ponz de Leon M (1997) Survival analysis in families affected by hereditary non-polyposis colorectal cancer. *Int J Cancer* **71**: 373–376

Perucho M (1999) Correspondence re: CR Boland et al, A National Cancer Institute Workshop on Microsatellite Instability for Cancer Detection and Familial Predisposition: Development of International Criteria for the Determination of Microsatellite Instability in Colorectal Cancer. *Cancer Res*, 58: 5248–5257, 1998. *Cancer Res* **59**: 249–253

Peto R, Pike M, Breslow N, Cox D, Howard S, Mantel N, McPherson K, Peto J and Smith P (1977) Design and analysis of randomized clinical trials requiring prolonged observations of each patient. 2. Analysis and example. *Br J Surg* **35**: 1–39

Sankila R, Aaltonen LA, Jarvinen HJ and Mecklin JP (1996) Better survival rates in patients with MLH1-associated hereditary colorectal cancer [see comments]. *Gastroenterology* **110**: 682–687

Tannergard P, Liu T, Weger A, Nordenskjold M and Lindblom A (1997) Tumorigenesis in colorectal tumors from patients with hereditary non-polyposis colorectal cancer. *Hum Genet* **101**: 51–55

Thibodeau S, Bren G and Schaid D (1993) Microsatellite instability in cancer of the proximal colon. *Science* **260**: 816–819

Thibodeau SN, French AJ, Roche PC, Cunningham JM, Tester DJ, Lindor NM, Moslein G, Baker SM, Liskay RM, Burgart LJ, Honchel R and Halling KC (1996). Altered expression of hMSH2 and hMLH1 in tumors with microsatellite instability and genetic alterations in mismatch repair genes. *Cancer Res* **56**: 4836–4840

Weber JL and May PE (1989) Abundant class of human DNA polymorphisms which can be typed using the polymerase chain reaction. *Am J Hum Genet* **44**: 388–396

Zhou XP, Hoang JM, Li YJ, Seruca R, Carneiro F, Sobrinho-Simoes M, Lothe RA, Gleeson CM, Russell SE, Muzeau F, Flejou JF, Hoang-Xuan K, Lidereau R, Thomas G and Hamelin R (1998) Determination of the replication error phenotype in human tumors without the requirement for matching normal DNA by analysis of mononucleotide repeat microsatellites. *Genes Chromosomes Cancer* **21**: 101–107

*© 1999 Cancer Research Campaign*
